# Supplementary figures and images for: Renal Transcriptome and Metabolome in Mice with Principal Cell-Specific Ablation of the Tsc1 Gene: Derangements in Pathways Associated with Cell Metabolism, Growth and Acid Secretion
Source: Int J Mol Sci. 2022 Sep 13;23(18):10601. doi: 10.3390/ijms231810601 (PMC9502912; doi:10.3390/ijms231810601)

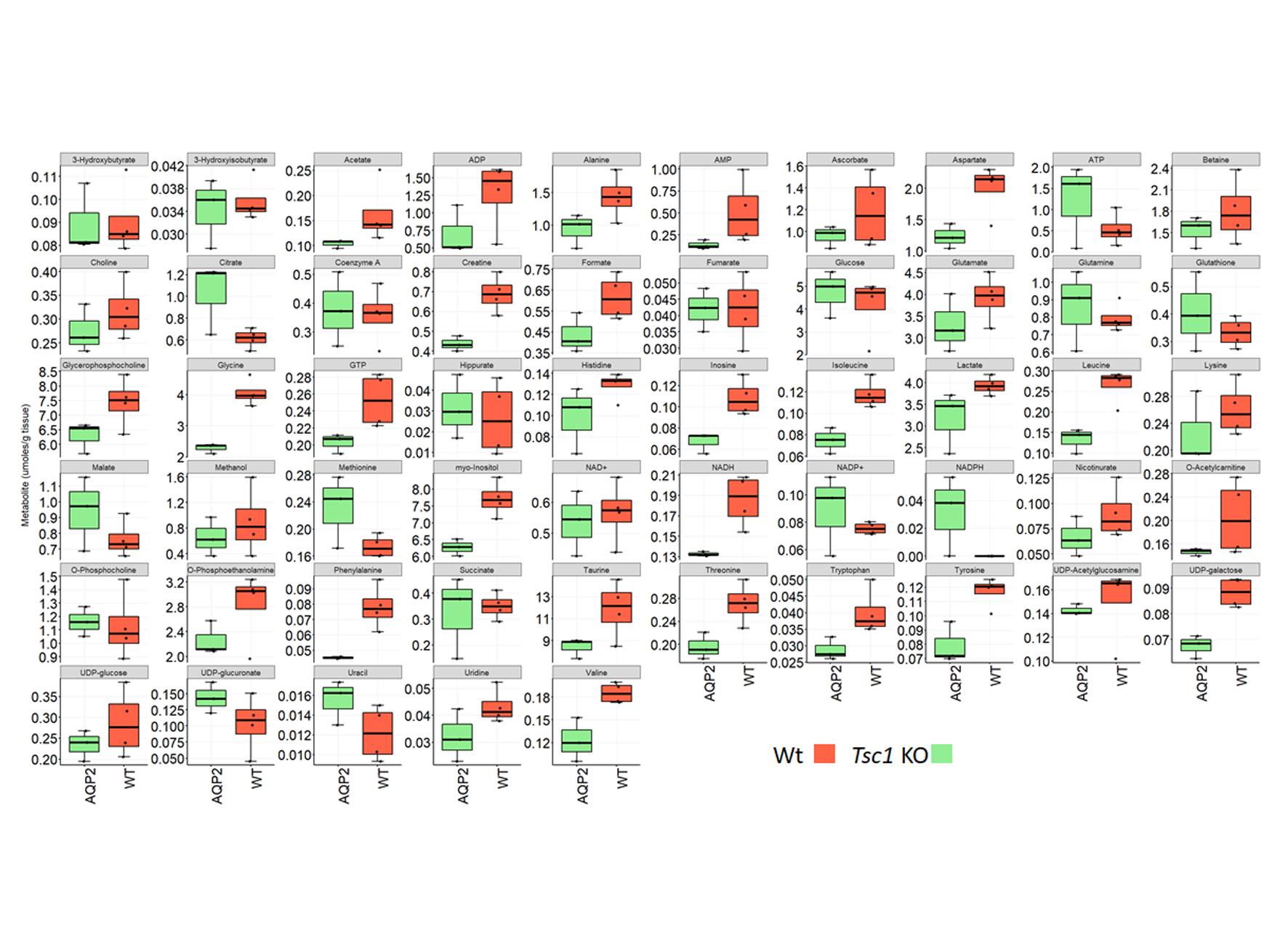

Supplement: Supplementary file 1 [file ijms-23-10601-s001.zip › Supplement Figure S1.jpg]

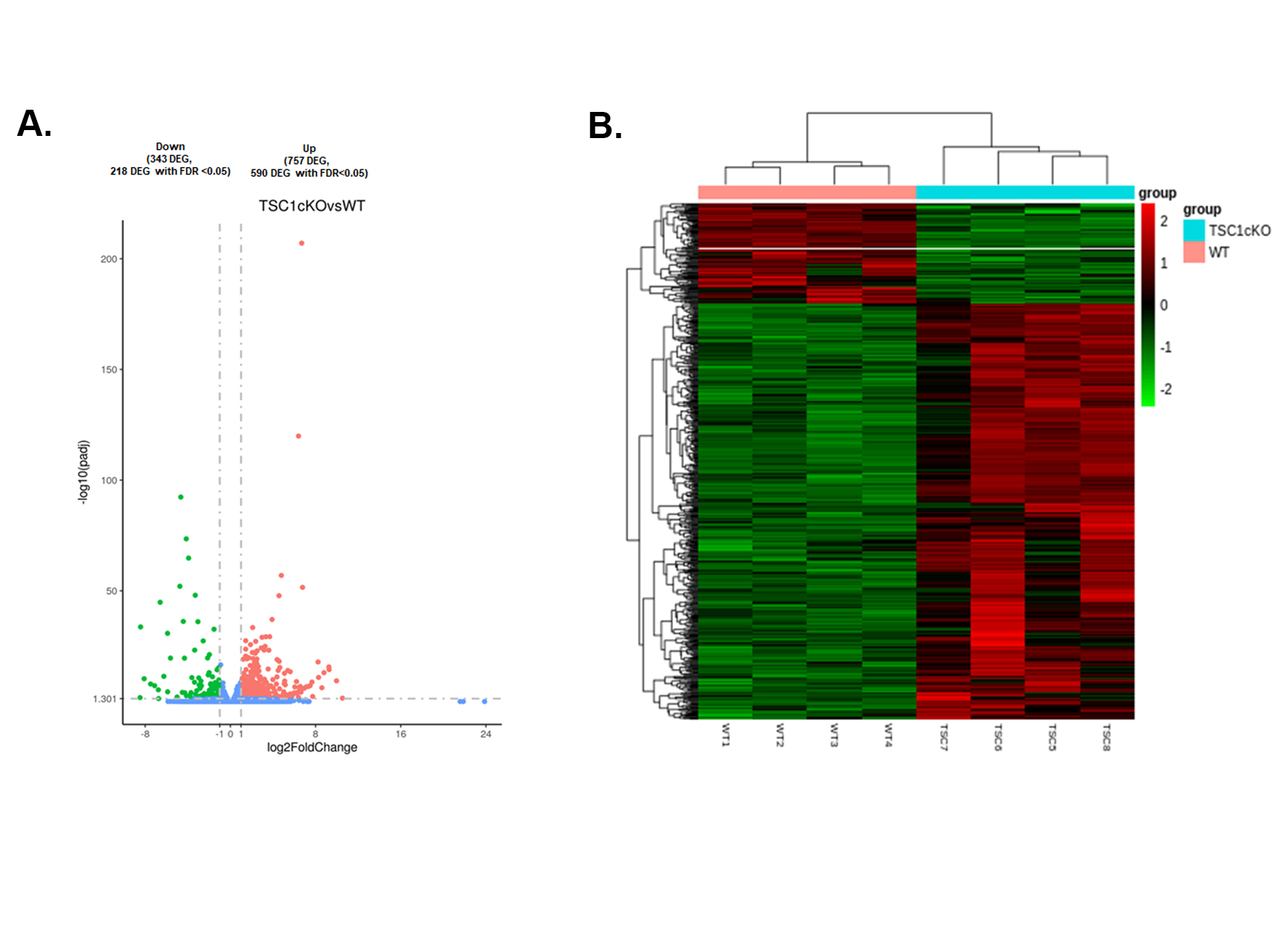

Supplement: Supplementary file 1 [file ijms-23-10601-s001.zip › Supplement Figure S2.jpg]

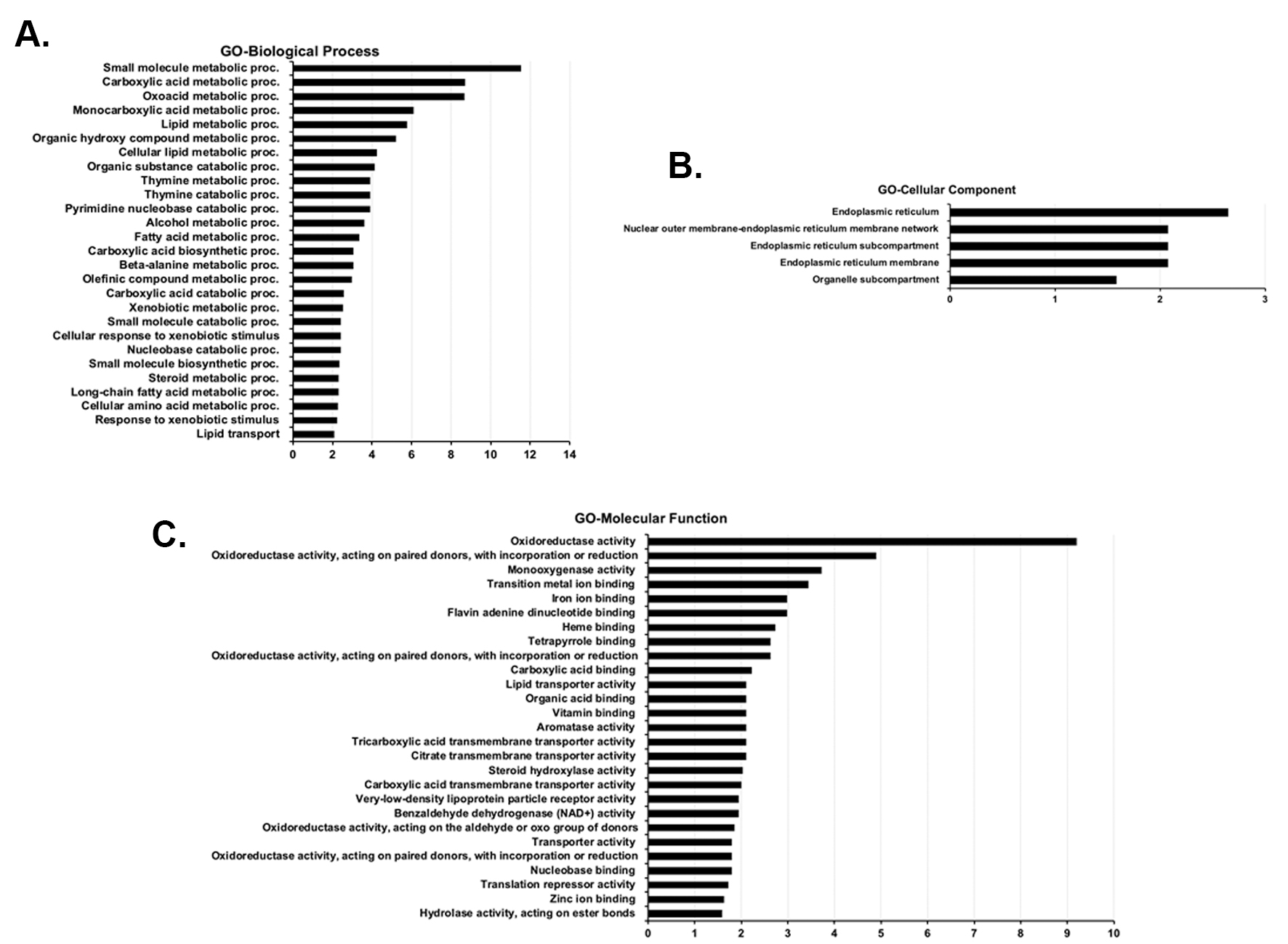

Supplement: Supplementary file 1 [file ijms-23-10601-s001.zip › Supplement Figure S3.jpg]

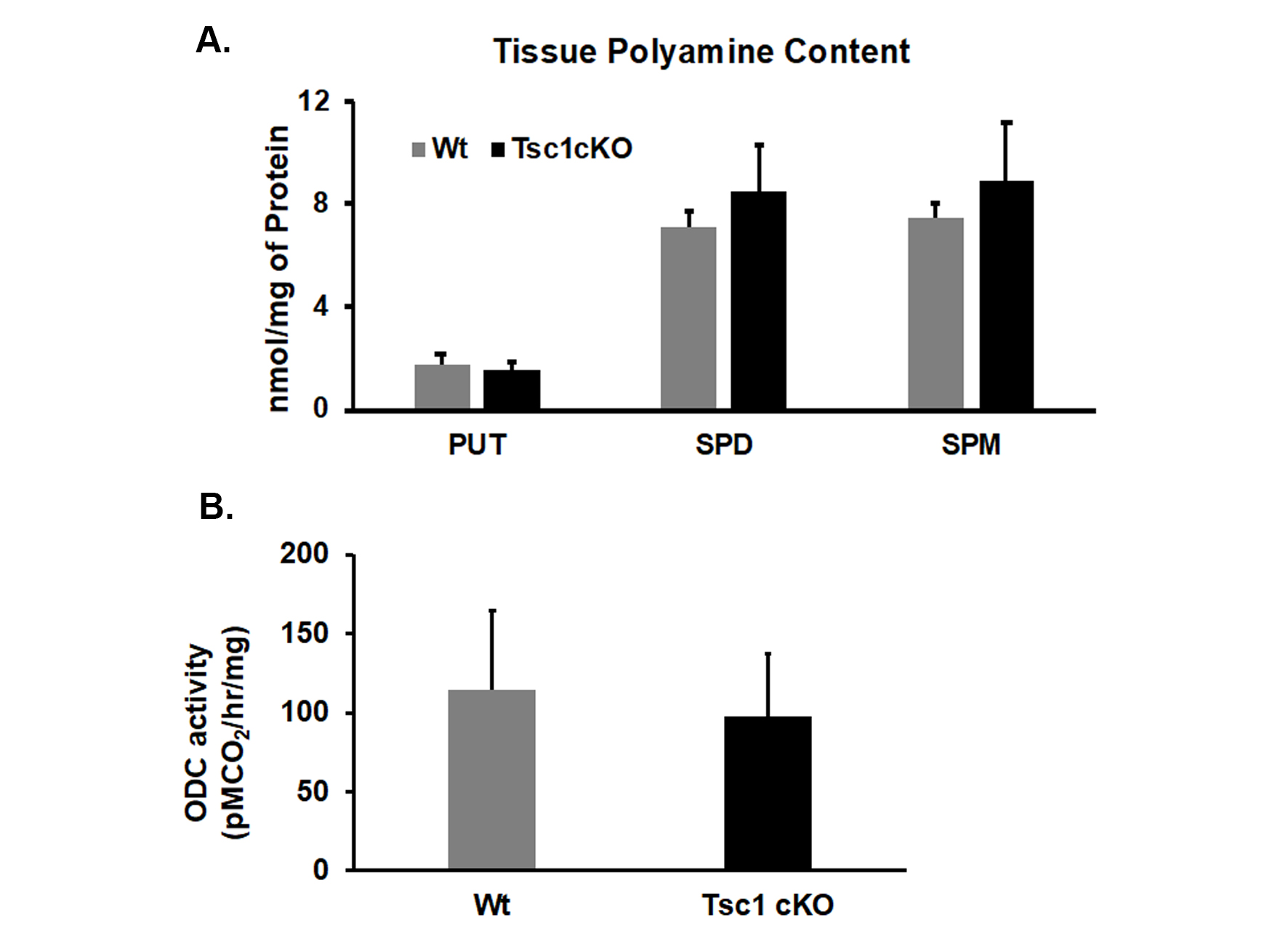

Supplement: Supplementary file 1 [file ijms-23-10601-s001.zip › Supplement Figure S4 .jpg]

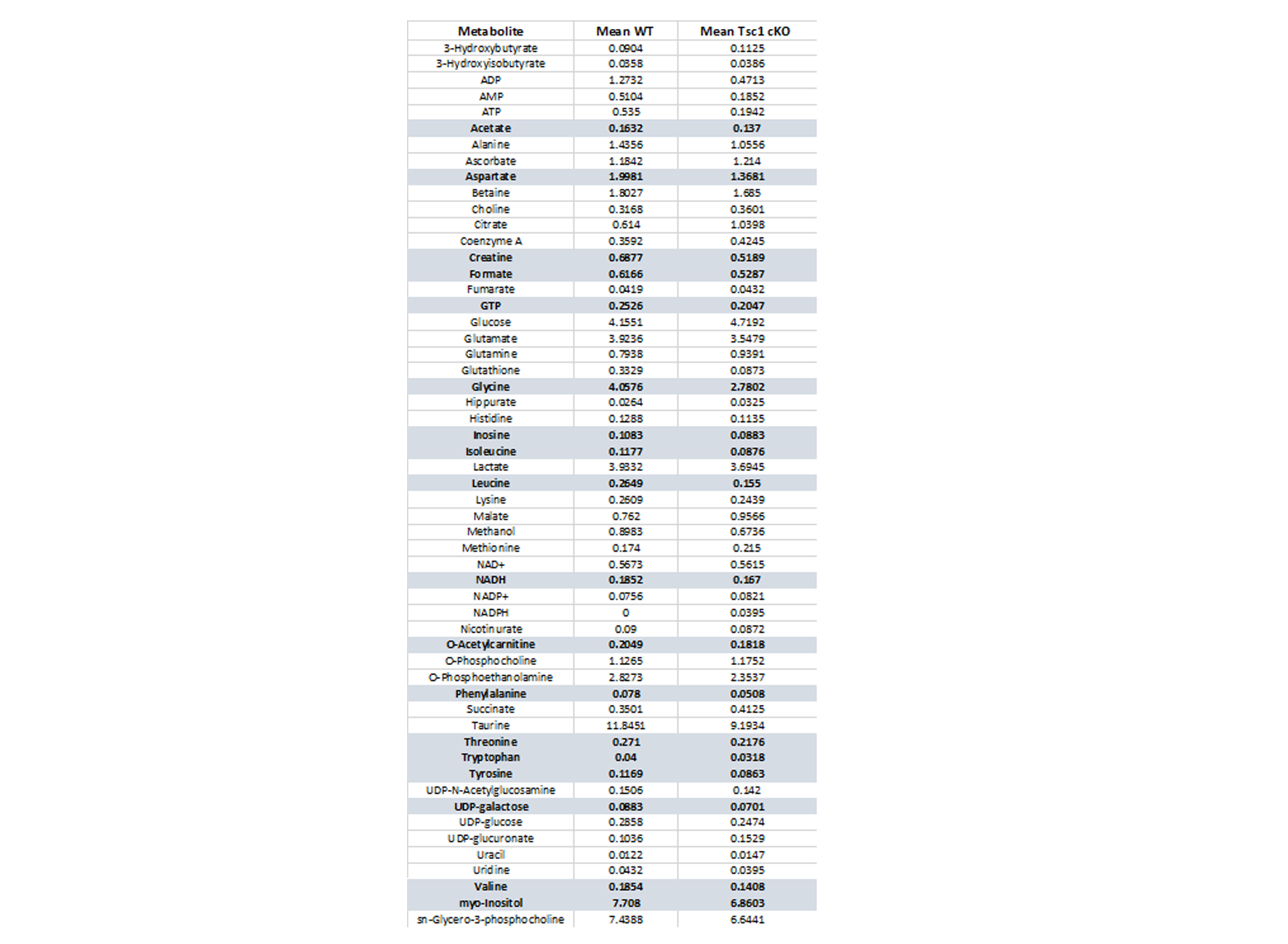

Supplement: Supplementary file 1 [file ijms-23-10601-s001.zip › Supplement Table S1 .jpg]
